# Transcriptomic Analysis of Rice Plants Overexpressing *PsGAPDH* in Response to Salinity Stress

**DOI:** 10.3390/genes12050641

**Published:** 2021-04-25

**Authors:** Hyemin Lim, Hyunju Hwang, Taelim Kim, Soyoung Kim, Hoyong Chung, Daewoo Lee, Soorin Kim, Soochul Park, Woosuk Cho, Hyeonso Ji, Gangseob Lee

**Affiliations:** 1 Department of Forest Bioresources, National Institute of Forest Science, Suwon 16631, Korea; supia1125@korea.kr (H.L.); ktlmi01@korea.kr (T.K.); 2 Department of Applied Marine Bioresource Science, National Marine Biodiversity Institute of Korea, Seocheon 33662, Korea; hjhwang@mabik.re.kr; 3 National Institute of Agricultural Science, Rural Development Administration, Jeonju 54874, Korea; sykimflower@korea.kr (S.K.); usdapark@korea.kr (S.P.); phyto@korea.kr (W.C.); jhs77@korea.kr (H.J.); 4 3BIGS CO. LTD., 156 Gwanggyo-ro, Suwon 16429, Korea; hychung@3bigs.com; 5 National Institute of Crop Science, Rural Development Administration, Suwon 16430, Korea; dlee@korea.kr; 6 School of Food Science & Biotechnology, Kyungpook National University, Daegu 41566, Korea; soorinkim@knu.ac.kr

**Keywords:** glyceraldehyde-3-phosphate dehydrogenase, salt stress, tolerance, transcriptomic analysis, transgenic rice

## Abstract

In plants, glyceraldehyde-3-phosphate dehydrogenase (GAPDH) is a main enzyme in the glycolytic pathway. It plays an essential role in glycerolipid metabolism and response to various stresses. To examine the function of PsGAPDH (*Pleurotus sajor-caju* GAPDH) in response to abiotic stress, we generated transgenic rice plants with single-copy/intergenic/homozygous overexpression *PsGAPDH* (*PsGAPDH*-OX) and investigated their responses to salinity stress. Seedling growth and germination rates of *PsGAPDH*-OX were significantly increased under salt stress conditions compared to those of the wild type. To elucidate the role of *PsGAPDH*-OX in salt stress tolerance of rice, an Illumina HiSeq 2000 platform was used to analyze transcriptome profiles of leaves under salt stress. Analysis results of sequencing data showed that 1124 transcripts were differentially expressed. Using the list of differentially expressed genes (DEGs), functional enrichment analyses of DEGs such as Gene Ontology (GO) terms and Kyoto Encyclopedia of Genes and Genomes (KEGG) pathways were performed. KEGG pathway enrichment analysis revealed that unigenes exhibiting differential expression were involved in starch and sucrose metabolism. Interestingly, *trehalose-6-phosphate synthase* (*TPS*) genes, of which expression was enhanced by abiotic stress, showed a significant difference in *PsGAPDH*-OX. Findings of this study suggest that PsGAPDH plays a role in the adaptation of rice plants to salt stress.

## 1. Introduction

Plants continuously face environmental stressors, including drought, high salinity, and extreme temperatures. These stressors affect both biomasses and grain yields of crops. Salt stress is a particularly important abiotic stress that can seriously affect the development, growth, and productivity of plants. Severe stress may threaten their survival. Rice (*Oryza sativa*) is a major staple food crop in the world. It is also a model plant for the genomics research of monocotyledons [1]. Salinity is one of the most disastrous abiotic stresses for rice. Salt-affected soils currently account for approximately 20% of global agricultural production [2]. Recently, many studies have reported molecular and cellular mechanisms such as ERF1, OsMYB91, and OsGLYII-2 involved in the tolerance of rice to salt [3,4,5]. It is well known that rice is extremely sensitive to salt stress. Thus, more genes might be involved in its salt resistance.

Glyceraldehyde-3-phosphate dehydrogenase (EC 1.2.2.12) (GAPDH) is a glycolytic enzyme that catalyzes the oxidation of triose phosphates during glycolysis in all organisms. GAPDH plays multiple roles in the regulation of autophagy, hypersensitive response, and plant immune responses [6,7,8]. In addition, the phosphorylation of NAD-GAPDH and comprehensive analysis of the *GAPDH* gene family in wheat were conducted [9,10]. GAPDH is an important enzyme in two metabolism pathways for sugar: glycolysis and gluconeogenesis. In plants, glycolysis is the predominant respiratory pathway that provides ATP, reductants, and precursors for plant growth and development [11]. Glycolysis is also involved in the adaptation of plants to stress conditions such as salt, cold, drought, and anoxia [12,13,14]. What is especially noteworthy is that salt stress tolerance can lead to the expression of *GAPDH* genes at the transcription level in fungi such as *Aspergillus nidulans* [15]. Based on this, we have isolated a *GAPDH* gene from the oyster mushroom, *Pleurotus sajor-caju (PsGAPDH)*, and characterized its expression under various abiotic stresses basis in a previous study [16]. Potato overexpressing *PsGAPDH* also shows increased salt tolerance [17]. However, no comprehensive physiological explanation has been provided. It is known that overexpression of genes in transgenic plants can improve plant resistance to various abiotic stresses [18]. In a previous study, we have generated transgenic rice plants overexpressing *PsGAPDH* [19]. To investigate the mechanism of *PsGAPDH* in salinity stress, transcriptome analysis was performed.

Lately, next-generation sequencing (NGS) technology has been advancing rapidly. It provides a fast, cost-effective, and comprehensive analysis of complex nucleic acid groups of model plants (or closely related species) and non-model plants [20,21]. Next-generation high-throughput RNA sequencing technology could overcome shortcomings of array-based technologies. With high resolution and sensitivity, RNA sequencing can be used to discover new splice junctions, new transcripts, alternative transcription start sites, and rare transcripts [22]. Additionally, RNA sequencing data show a high level of reproducibility in both technical and biological replications [23]. To date, global gene expression profiles of organisms including plants have been obtained by RNA sequencing [24,25,26,27]. Moreover, RNA sequencing has been widely used in comparative transcriptomics to recognize differences in transcript abundance among different cultivars, organs, and treatment conditions [28]. To identify genes exhibiting transcriptional changes, we compared transcriptomes of salt-stressed and control rice plants and analyzed their functions and KEGG pathways.

In this study, we used RNA sequencing to analyze transcriptomes of wild-type and *PsGAPDH*-overexpressing rice plants under salinity stress to obtain detailed expression profiles of genes involved in salt stress response. Assembled and annotated transcriptome sequences and their transcription abundance patterns will provide a valuable genetic resource to further investigate molecular mechanisms involved in the salt tolerance of rice. In particular, among candidates for this genetic resource, the *TPS* genes showed a significant difference in expression in *PsGAPDH*-OX. *OsTPS1* has been reported to increase the expression by abiotic stress and enhance the expression of stress-related genes [29]. These results suggested that PsGAPDH may play a role in tolerance mechanisms of salinity stress.

## 2. Materials and Methods

### 2.1. Plant Materials and Generation of Transgenic Rice

Rice (*Oryza sativa* L. cv. Dongjin) was used in this study. Sterilized seeds were germinated on MS medium in a growth chamber equipped with fluorescent lamps (28 °C, 16 h light/8 h dark cycle, 300 μmol m^−2^ s^−1^). Dongjin seeds were used to produce *PsGAPDH*-overexpressing rice plants. Rice seeds were sterilized with 70% ethanol and 2% NaClO. All tissue samples collected were immediately frozen in liquid nitrogen and stored at −70 °C.

**Figure 1:**
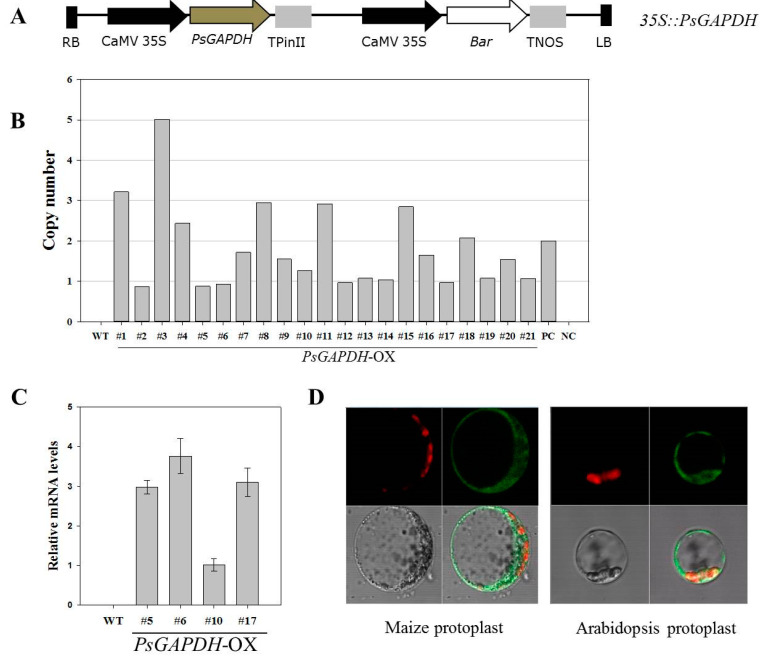
Generation of transgenic rice plants overexpressing *PsGAPDH*. (**A**) Schematic diagram of a plant expression vector pPZP-*PsGAPDH* used for overexpressing *PsGAPDH* in transgenic rice. (**B**) TaqMan copy number analysis of *PsGAPDH* transgenic plants for transgene copy determination. (**C**) Expression levels of *PsGAPDH* in transgenic plants by quantitative real time-PCR. Three transgenic lines (#5, #6, and #17) displayed high *PsGAPDH* expression compared to #10 plant. (**D**) Subcellular localization of PsGAPDH. Fluorescence signals in maize and Arabidopsis protoplasts were visualized using a confocal laser-scanning microscope. #, number; RB,T-DNA right border; LB, T-DNA left border; WT, wild type; PC, positive control; NC, negative control.

To construct an overexpression vector, full-length cDNA of *PsGAPDH* was cloned into a plant expression vector pPZP, which was constructed by introducing *CaMV35S* promoter and *PinII* terminator containing *Bar* as a plant selectable marker (Figure 1A) [19,30]. The *35S::PsGAPDH* construct was introduced into an *Agrobacterium tumefaciens* strain LB4404 to generate transgenic rice [19]. Rice plants were transformed by *Agrobacterium* infection as described previously [31]. Transgenic rice plants were regenerated from transformed calli on selection medium containing 4 mg/L phosphinothricin and 500 mg/L cefotaxime and subsequently grown in a greenhouse.

### 2.2. Genomic DNA Extraction and TaqMan Copy Number Analysis

Genomic DNA of transgenic plants was extracted using an IncloneTM Genomic DNA Prep Kit (Inclone, Korea). Copy number assay was performed on an Applied Biosystems StepOnePlusTM (Applied Biosystems, Foster City, CA, USA) with a TaqMan^®^ Gene Expression Master Mix (Applied Biosystems) kit. Primers and probes of the pre-designed TaqMan^®^ copy number assay for rice *tubulin α-1 chain* gene (AK102560) as an endogenous control were used. For the transgene, primers and probes were specifically designed for the terminator of the *nos* gene. Gene-specific primers used were NOS-F 5′-GCATGACGTTATTTATGAGATGGGTTT-3′ and NOS-R 5′-TGCGCGCTA TATTTTGTTTTCTATCG-3′. NOS-probe 5ʹ-TAGAGTCCCGCA ATTAT-3′ was also used for the *nos* gene. PCR conditions were as follows: 10 min at 95 °C; followed by 40 cycles of 95 °C for 15 s, 60 °C for 1 min, and 72 °C for 1 min. In the reaction plate, each sample was measured in triplicate. To calculate the copy number of a target gene, relative quantitation analysis of genomic DNA target was analyzed based on real-time PCR data using Applied Biosystems CopyCaller^®^ Software v2.0 (Applied Biosystems) according to the manufacturer’s instructions.

### 2.3. Transient Expression Analysis in Protoplast

The cDNA sequence of *PsGAPDH* was cloned with a GFP fusion protein (*PsGAPDH:GFP*) into a pJJ2485-GFP expression vector using a gateway recombination system (Invitrogen, Life technologies, Carlsbad, CA, USA). Protoplast transformation was performed as previously described [32]. Transformed protoplasts were observed with a confocal microscope (LSM 510 META, Carl Zeiss, Jena, Germany). Autofluorescence of chlorophyll was used as a chloroplast marker.

### 2.4. Salt Stress Treatment

Salt stress treatment for transgenic rice plants was performed using a published method [33]. For the phenotypic analysis of *PsGAPDH*-OX plants in response to salt stress, sterilized rice seeds were transferred into 1/2 Murashige and 1/2 MS medium containing 100 mM NaCl or 1/2 MS liquid medium containing 200 mM NaCl. Seeds were incubated at 28 °C for 5 days or 14 days. Germination and seedling growth of transgenic plants and wild-type seeds sown on MS agar plates at each nutrient level was monitored. Three replicates with 15 seeds in each replicate were used. All data were analyzed by *t*-tests. Significant differences are indicated by asterisks (*, *p <* 0.05; **, *p <* 0.01). All the data from control and treatments were subjected to statistical analysis using SPSS 20.0.

For RNA sequence analysis of *PsGAPDH*-OX in response to salt stress, two-week-old seedlings were transferred to soil culture in a greenhouse. After 7 weeks, all plants were subjected to treatment with 200 mM NaCl (salt stress) for 3 days. RNA sequence analyses of three biological replicates for each wild-type and transgenic plants were then performed.

### 2.5. RNA Extraction, cDNA Library Construction, and Sequencing

Total RNAs were extracted from rice leaves using an RNeasy^®^ Plant mini kit (QIAGEN, Hilden, Germany) according to the manufacturer’s protocol. RNA quality was determined with a 2100 Bioanalyzer (Agilent, Santa Clara, CA, USA). Only samples with an RNA integrity number >8 were used for library preparation. Each paired-end cDNA library was constructed using a TruSeq RNA Sample Preparation Kit (Illumina, San Diego, CA, USA).

### 2.6. Pre-Processing of RNA Sequencing Data

Based on RNA libraries generated, paired-end sequencing was performed using a Hiseq2000 platform (Illumina). To generate clean reads, Trimmomatic (ver 0.36; [34]) was used. Per-base sequence qualities were checked using FastQC (ver. 0.11.2) (http://www.bioinformatics.babraham.ac.uk/projects/fastqc/ accessed on 26 March 2021). Fastq files were then filtered. Trimmed reads were aligned to a reference genome (Oryza_sativa.IRGSP-1.0) using HISAT software version 2-2.1.0 [35]. To compare reads containing the strand information to those without, reads were also aligned without using the ‘--RNA-strandness RF’ option. We quantified mapped reads using FeatureCounts with annotation files (.GFF3) for protein-coding genes [36].

### 2.7. Differential Expressed Gene (DEG) Analysis

Differentially expressed genes (DEGs) were identified using an edgeR Bioconductor package based on a Generalized Linear Model (GLM) used for RNA-seq data analysis, considering gene expression as a negative binomial [37]. The EdgeR-robust method [38] was used to reduce the effect of outlier genes. Based on quantified gene expression, relationships among samples and stages were investigated using a multidimensional scaling (MDS) method. A heatmap was generated to visualize DEGs which included the 500 most expressed genes (TMM upper value) across samples using R. A volcano plot was obtained to visualize differential expression between samples. Significant differences (Log-fold change > 2.0 or Log-fold change < −2.0, *p*-value < 0.05) between two different conditions were illustrated in the volcano plot. All identified proteins were annotated with the Os-Nipponbare-Reference-IRGSP-1.0 database (https://rapdb.dna.affrc.go.jp accessed on 26 March 2021). An adjusted *p*-value (FDR) < 0.05 was used as the significance cutoff for differentially expressed genes.

### 2.8. Gene Ontology (GO) and Kyoto Encyclopedia of Genes and Genomes (KEGG) Pathway Analysis

DEGs were further processed with DAVID 6.8 Functional Annotation Tool (http://david.abcc.ncifcrf.gov/ accessed on 26 March 2021) for term enrichment analysis [39]. Results were filtered using a Fisher’s exact statistic methodology as previously described [39].

### 2.9. Quantitative Real-Time PCR (qRT-PCR)

Transcriptome sequencing data were validated by qRT-PCR. Briefly, RNAs (1 µg) were reverse-transcribed to cDNAs using an AmfiRevert cDNA synthesis kit (Gendepot, USA). Synthesized cDNAs were used as templates for qRT-PCR. Primer pairs specific for the amplification of target genes are shown in Supplementary Table S4. Relative quantification was performed to calculate expression levels of target genes in different treatments using the 2^−∆∆Ct^ method. The expression level of OsActin1 was used for the normalization of real-time PCR results. All data are expressed as the mean ± SD from three independent experiments.

## 3. Results

### 3.1. Generation of Rice Plants Overexpressing PsGAPDH

The *PsGAPDH* gene from *Pleurotus sajor-caju* (*Oyster mushroom*) is highly induced by salt, drought, cold, and heat stress conditions [16,17]. Interestingly, it has been reported that transgenic potato plants constitutively expressing the *PsGAPDH* gene can increase their salt tolerance under salt stress conditions [17]. Thus, rice plants overexpressing *PsGAPDH* (*PsGAPDH*-OX) were generated by introducing a construct containing the full-length cDNA of *PsGAPDH* under the control of a *CaMV35S* promoter (Figure 1A). A total of 149 T0 generation lines of *PsGAPDH*-OX rice plants were generated. TaqMan copy number assay was used to select single-copy lines from *PsGAPDH*-OX plants (Figure 1B). Flanking sequence tag (FST) analysis of T0 plants was carried out only for those transgenic plants in which a single copy T-DNA insertion was confirmed. Transgenic plants with the T-DNA inserted in an intergenic region are less likely to be affected by other genes. Hence, single copy/intergenic transgenic plants were selected using FST analysis (Supplementary Table S1) [19]. We obtained five single copy/intergenic transgenic plants from 21 lines of transformants. *PsGAPDH*-OX (#5, #6, #10, and #17) plants with a single-copy and an intergenic insertion were selected and separated to produce homozygous T3 generation. Transcriptional levels of the *PsGAPDH* gene in these four lines of single-copy/intergenic/homozygous *PsGAPDH*-OX plants were determined using qRT-PCR. Results of qRT-PCR revealed that *PsGAPDH* was overexpressed in transgenic rice plants. *PsGAPDH*-OX #10 line showed a relatively low level of expression compared to other transgenic rice lines (Figure 1C). Single-copy/intergenic/homozygous *PsGAPDH*-OX (#5, #6, and #17) plants were then selected and used in subsequent experiments.

### 3.2. Subcellular Localization of PsGAPDH

GAPDH catalyzes the conversion of glyceraldehyde-3-phosphate to 1,3-bisphospho glycerate. Both cytosolic (GAPCs) and plastidial (GAPCps) GAPDH activities have been described in plants. To determine the subcellular localization of *PsGAPDH* expression, the distribution of *PsGAPDH* in maize and Arabidopsis protoplasts was examined. PsGAPDH–GFP proteins were transiently expressed in protoplasts isolated from leaves using a polyethylene glycol (PEG)-mediated transfection system [32]. A fusion protein with GFP was expressed and its localization was visualized using a laser scanning confocal microscope. As shown in Figure 1D, PsGAPDH–GFP was predominantly present in the cytoplasm of Arabidopsis and maize. This result demonstrates that PsGAPDH is protein-localized in the cytoplasm.

### 3.3. Effect of PsGAPDH-OX on Seedling Growth under Salt Stress Condition

**Figure 2:**
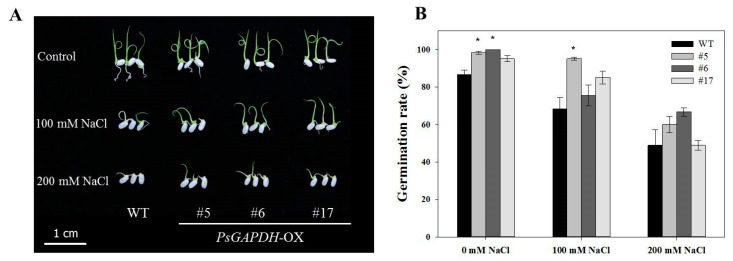
Seed germination rate of *PsGAPDH*-OX transgenic plants under salt stress. (**A**) Seed germination phenotypes of wild-type (Control) and *PsGAPDH*-OX plants treated with 100 mM or 200 mM NaCl for 5 days. (**B**) Graph of germination rates. For each treatment, 15 seeds were measured. Error bar represents the SD of three replicates. Significant differences depending on *t*-tests are indicated by asterisks (*, *p* < 0.05).

**Figure 3:**
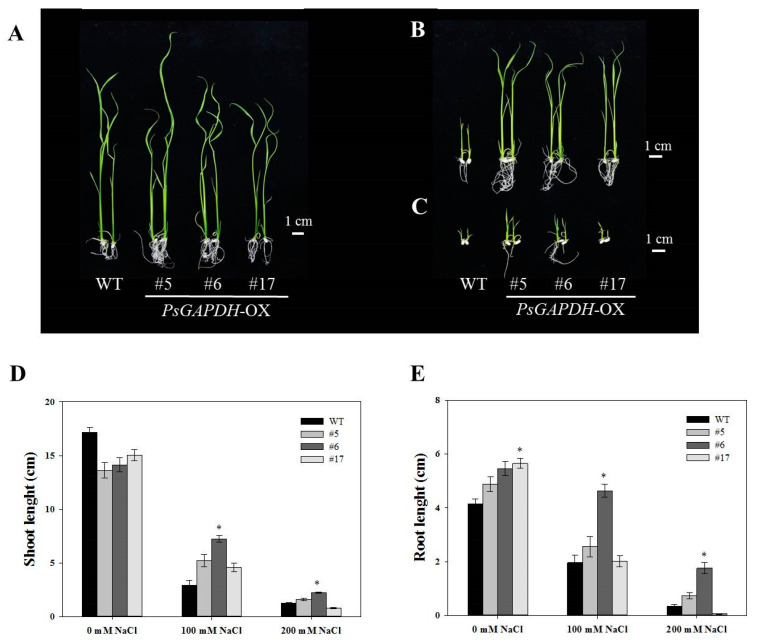
Phenotypic comparison of *PsGAPDH*-OX transgenic plants under salt stress. Phenotypes of wild-type and *PsGAPDH*-OX rice plants grown on MS medium containing 0 mM (**A**), 100 mM (**B**), and 200 mM (**C**) NaCl for 2 weeks. Effect of 2 weeks of salt stress on shoot (**D**) and root (**E**) lengths of *PsGAPDH*-OX plants. For each treatment, 15 seedlings were measured. Error bar represents the SE of three replicates. Significant differences depending on *t*-tests are indicated by asterisks (*, *p* < 0.05).

To assess the role of PsGAPDH in the salt stress response of rice, seeds of PsGAPDH-OX and wild-type rice were sown on MS agar plates containing different concentrations (100 mM and 200 mM) of NaCl. Their germination rates and seedling growths were monitored. There was no significant difference between the wild-type and PsGAPDH-OX cultured in MS medium without NaCl (Figure 2). However, when they were cultured in MS medium containing 100 mM or 200 mM NaCl for 8 days, PsGAPDH-OX #5, #6, and #17 rice lines showed different germination rates from that of the wild-type. In MS medium containing 100 mM NaCl, the germination rate of wild-type rice seeds was 68%, while those of PsGAPDH-OX #5, #6, and #17 were 95%, 76%, and 85%, respectively (Figure 2). In MS medium containing 200 mM NaCl, the germination rate of the wild-type rice seed was 49%, while those of PsGAPDH-OX #5, #6, and #17 were 60%, 67%, and 49%, respectively. After 14 days of culturing, PsGAPDH-OX #6 seedlings showed significantly better growth performance than the wild-type (*p* < 0.05) on MS media supplemented with 100 mM or 200 mM NaCl (Figure 3). These results suggested that PsGAPDH-OX lines were resistant to salt stress.

### 3.4. Statistical Test for DEG

**Figure 4:**
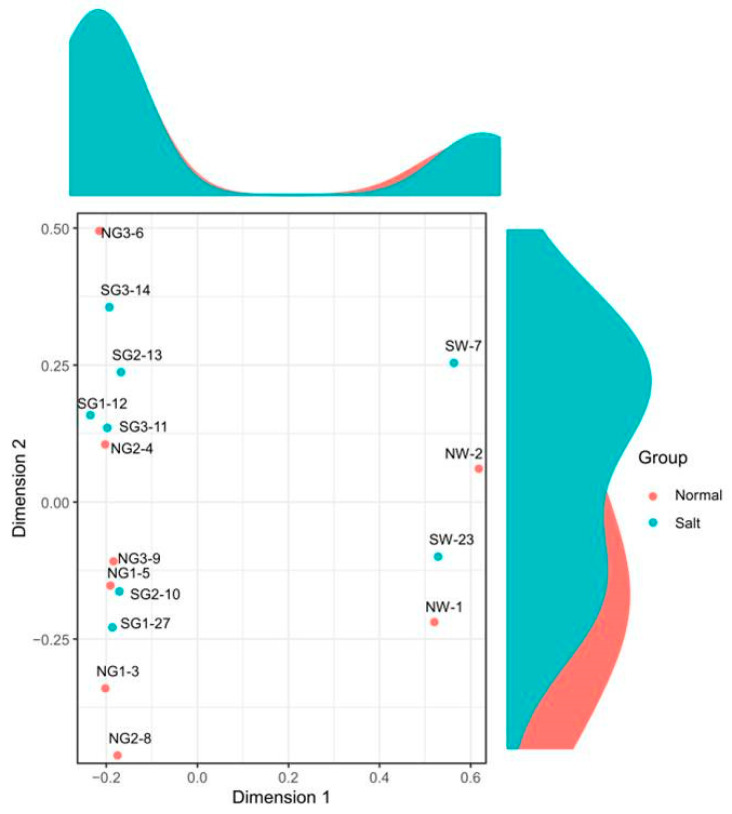
Multi-dimensional scaling (MDS) plot based on the transcriptomic analysis of wild-type rice plants and transgenic rice plants overexpressing *PsGAPDH*. Each point represents an RNA-seq sample. “Normal” and “Salt” groups are illustrated.

**Figure 5:**
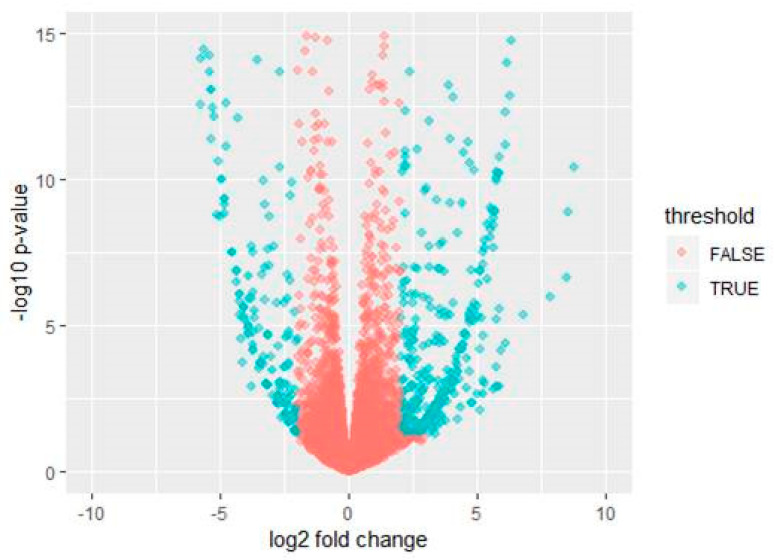
Expression changes of all genes. The volcano map displays expression changes of all genes. Blue represents differentially expressed genes (Log-fold change > 2.0 or Log-fold change < −2.0, *p*-value < 0.05) between Normal and *PsGAPDH* transgenic samples.

Based on quantified gene expression, relationships among samples and stages were investigated using the MDS method. Multi-dimensional plot indicated that samples could be distinguished by transgenic rice (Figure 4). After checking distances of samples, statistical tests were performed to identify DEGs between control and transgenic samples. Total 1124 genes expressed significant differences that up-regulated and down-regulated genes were 603 and 521, respectively (Figure 5).

**Figure 6:**
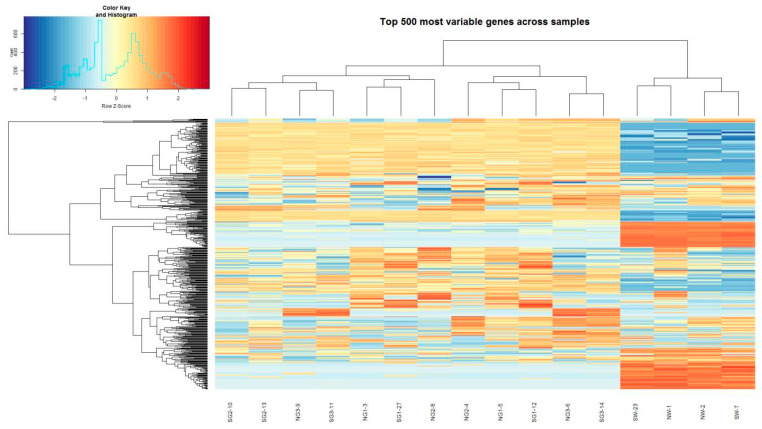
Expression patterns of the most significantly expressed genes across total samples. In the heatmap, the color indicates the expression level of a gene and the column header indicates a sample.

The top 10 down-regulated DEGs and top 10 up-regulated DEGs matched with Oryzabase gene symbols and Oryzabase gene name synonym(s) with an adjusted *p*-value < 0.05 (Table 1). Typically, many F-box protein genes were up- or down-regulated. A total of 396 F-box genes were included in 1124 DEGs (Supplementary Table S3). The heatmap of the 500 most expressed genes (TMM upper value) across samples showed that control samples were clustered with each other (Figure 6). A total of 1124 DEGs were selected based on a significance cutoff of FDR < 0.05.

### 3.5. GO and KEGG PATHWAY Analysis

**Figure 7:**
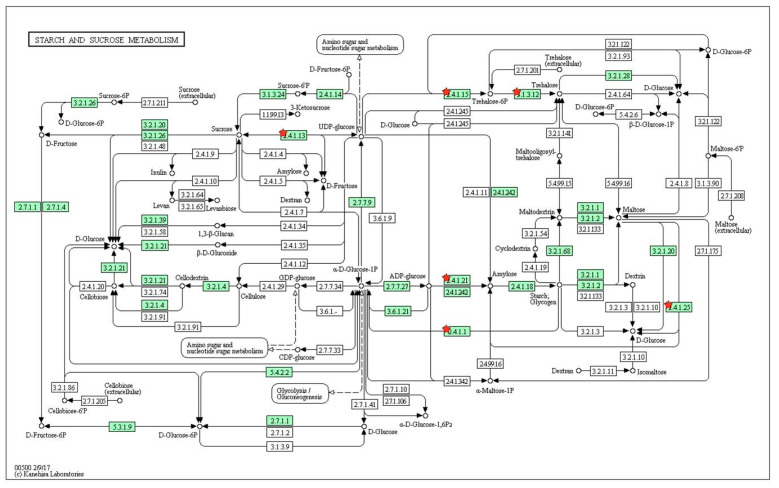
KEGG pathway for starch and sucrose metabolism. Four genes (*DPE2*, *SS2*, *TPS3*, and *SUS2*) are marked in red. We identified the metabolism pathway using DAVID.

Using the list of 1124 DEGs, functional enrichment of DEGs such as GO terms and the KEGG pathway were investigated (Table 2). In KEGG pathways of DEGs, metabolism of starch and sucrose was significantly enriched (Figure 7). Trehalose-6-phosphate (T6P), an intermediate of trehalose biosynthesis, is an essential signal metabolite in plants. It is linked to growth and development according to carbon status. In this study, the *trehalose-6-phosphate synthase* (*TPS*) gene for transferring Gluc-6P to T6P was down-regulated, whereas the *trehalose-6-phosphate phosphatase* (*TPP*) gene for transferring T6P to trehalose was up-regulated (Table 3). After TPS converts glucose-6-phosphate and UDP-glucose into T6P, T6P is then dephosphorylated into trehalose by TPP.

### 3.6. Verification of Differential Expression by Quantitative Real-Time PCR

**Figure 8:**
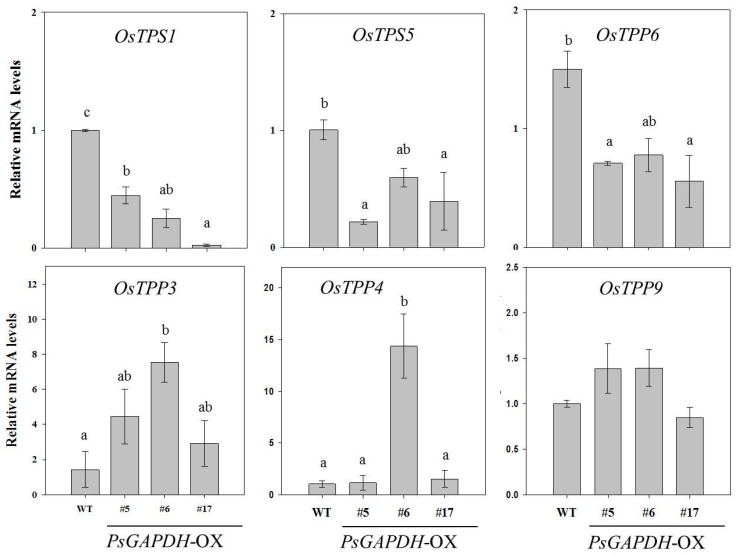
Expression levels of starch and sucrose-associated genes in wild-type and *PsGAPDH*-OX plants under NaCl stress conditions. Quantitative real-time PCR data were analyzed using the 2^−∆∆CT^ method with *OsActin1* gene as an internal control. Values are presented as means ± SD of three independent measurements. Different lowercase letters indicate significant differences (ANOVA with Tukey’s HSD, *p* < 0.05).

To validate expression profiles of genes in RNA sequencing results, we performed qRT-PCR for two *trehalose-6-phosphate synthase* (*TPS*) genes, *OsTPS1* (Os11t0156600) and *OsTPs5* (Os02t0790500), and four for *trehalose-6-phosphate phosphatase* (*TPP*) genes, *OsTPP6* (Os08t0409100), *OsTPP3* (Os09t0332100), *OsTPP4* (Os02t0753000), and *OsTPP9* (Os03t0386500). Expression levels of *OsTPS1* and *OsTPS5* genes were decreased in *PsGAPDH*-OX plants compared to those in the wild-type. Furthermore, the transcript levels of the *TPP* genes were significantly induced (Figure 8). Results of qRT-PCR correlated well with transcription levels estimated from RNA sequencing data, thus supporting the involvement of these genes in the defense against salinity stress in transgenic rice plants. Additionally, these results indicated that low levels of T6P and high levels of trehalose in *PsGAPDH*-OX rice plants might play a role in the resistance of transgenic plants against abiotic stress.

## 4. Discussion

*GAPDH* genes are commonly used as internal controls for the relative quantitation of gene expression [40]. However, a recent study has revealed that GAPDH displays unacceptably high expression variability, thus limiting its use as an internal control [41]. Indeed, several *GAPDH* genes have been characterized in relation to abiotic stresses. In particular, transgenic rice plants overexpressing *OsGAPC3* show significantly increased tolerance to salt stress, both during and after germination [40]. In the present study, we found that *PsGAPDH*-OX rice plants were more adaptive to salt stress than wild-type ones at germination and seedling growth stages (Figure 2 and Figure 3). Especially, *PsGAPDH*-OX #6 was the most tolerant against salt stress. This result suggests that GAPDH activity is required for the acclimation of rice plants to salt stress in the early growth stage. GAPDH is a highly conserved glycolytic enzyme that plays an important role in carbon metabolism pathways. Glycolysis is a principal metabolic pathway that provides precursors for biosynthetic reactions and takes part in the stress adaptation of plants [12,13,14]. Recent metabolomics analyses have demonstrated that long-term salt stress can alter cellular metabolic processes, including glycolysis, gluconeogenesis, and sucrose metabolism [13,40]. Plants can accumulate osmotic substances such as proline, trehalose, and sucrose to adapt to salt stress, because they can induce metabolic processes associated with osmolyte production [13,40].

Our RNA sequencing analysis data indicated that overexpression of *PsGAPDH* caused transcriptomic changes for a considerable number of genes involved in various physiological processes of rice. Those genes found to be responsive to salt stress in rice plants overexpressing *PsGAPDH* in the present study have previously been reported to be involved in trehalose biosynthesis processes such as *trehalose-6-phosphate synthase* (*TPS*) and *trehalose-6-phosphate phosphatase* (*TPP*). Among various osmolytes, trehalose (α-D-glucopyranosyl-α-D-glucopyranoside), a non-reducing disaccharide, is a principal compatible solute and a potential signal metabolite in plants. In plants, there is only one pathway for trehalose biosynthesis. It consists of a two-step process involving TPS and TPP that synthesize and subsequently dephosphorylate trehalose-6-phosphate (T6P) as an intermediate. Previous studies have shown that genetic modification of the *TPS/TPP* gene can increase stress resistance in different species [42,43]. Heterologous expression of bacterial or yeast trehalose biosynthesis gene in tobacco, Arabidopsis, rice, and potato can increase their stress tolerance [44]. Overexpression of *OsTPP1* can improve the resistance to salt and cold. It can also trigger the expression of a series of abiotic response genes, thus increasing the stress tolerance of rice [45]. Previous studies have shown that some *TPS/TPP* genes are directly involved in stress tolerance by improving trehalose contents in several plants [46].

## 5. Conclusions

Our results suggest that the *PsGAPDH* gene might participate in salt tolerance through coordination with these responsive genes. Hence, we speculate that *PsGAPDH* is possibly involved in various cellular processes in addition to the metabolism of starch and sucrose. Taken together, our findings suggest that PsGAPDH plays an important role in the metabolism of starch and sucrose during the adaptation of plants to salt stress.

## Figures and Tables

**Table 1:** Log-fold changes of top 10 down-regulated DEGs and top 10 up-regulated DEGs in Normal sample vs. *PsGAPDH* transgenic sample. Table 1 contains top 10 down-regulated DEGs and top 10 up-regulated DEGS with an adjusted *p*-value < 0.05.

Genes	Gene Symbol	Gene Name	logFC	FDR
**LogFC < 0**				
Os11t0532600-01	OsFbox601	F-box protein 601	−8.756393	1.61 × 10^−147^
Os10t0138100-01	AO	aldehyde oxidase	−8.680868609	3.26 × 10^−90^
Os12t0247700-01	OsJAC1, JAC1	jacalin-related lectin 1	−5.864641	1.82 × 10^−17^
Os11t0691800-01	OsRLCK354	Receptor-like Cytoplasmic Kinase 354	−5.791724	1.03 × 10^−12^
Os11t0615800-01	OsRAD51A1	DNA repair protein RAD51A1	−5.684418	5.72 × 10^−23^
Os07t0535100-01	OsFbox375	F-box protein 375	−5.682947	1.10 × 10^−14^
Os12t0420000-00	OsSTA280		−5.490965	2.02 × 10^−80^
Os12t0221600-01	OsRALF-35	Rapid alkalization factor 35	−5.368038	1.10 × 10^−11^
Os11t0685700-00	OsWRKY61	Rice WRKY gene61	−5.264582	8.04 × 10^−69^
Os01t0824700-01	OsFbox048	F-box protein 48	−4.959153931	5.93 × 10^−72^
**LogFC > 0**				
Os12t0121600-01	OsCHX09	cation/H+ exchanger 9	7.468688	2.79 × 10^−21^
Os02t0825500-01	OsFbox123	F-box protein 123	7.421409	1.78 × 10^−24^
Os02t0771600-01	OsACO3	ACC oxidase 3	7.030725	1.27 × 10^−43^
Os07t0158900-01	OsFbox341	F-box protein 341	6.116181	7.87 × 10^−46^
Os06t0286700-00	Piz(Pi2, Pi-z, Piz)	Pyricularia oryzae resistance-z, Magnaporthe grisea resistance-z	4.353849	1.65 × 10^−27^
Os12t0601400-01	OsIAA3	Aux/IAA protein 3, Aux/IAA protein 31	3.131579	1.36 × 10^−16^
Os12t0577700-00	Orysa; CKL1	CDK-LIKE 1, cyclin-dependent kinase-like 1	2.877962	3.72 × 10^−26^
Os11t0533800-01	OsFbox602	F-box protein 602	2.200298	4.17 × 10^−30^
Os10t0136500-01		receptor-like kinase	2.18271271	2.30 × 10^−16^
Os10t0437100-01	OsCAT7	cationic amino acid transporter 7	1.511570768	2.80 × 10^−17^

**Table 2:** Functional classification of differentially expressed proteins in rice samples. Proteins are classified into three main categories by GO analysis: biological process, cellular component, and molecular function. Column headers indicate the number of genes in a category, the percentage of a specific category of genes in that category, and the name of genes in that category. KEGG analysis showed one metabolism category. Results were filtered with a *p*-value < 0.05 as a cutoff.

GO Term	Count	Percent of Proteins	*p*-Value	Genes
**Biological Process**				
GO:0051365~cellular response to potassium ion starvation	2	4.57%	0.015	CIPK9, SS2
GO:0010555~response to mannitol	2	4.57%	0.017	CIPK9, SUS1
GO:0030154~cell differentiation	4	9.15%	0.020	FT, PI, MYB61, MYB55
GO:0055062~phosphate ion homeostasis	2	4.57%	0.027	CAX1, PHO2
GO:0051026~chiasma assembly	2	4.57%	0.031	MSH4, DMC1
GO:0009684~indoleacetic acid biosynthetic process	2	4.57%	0.033	NIT1, AMI1
**Cellular Component**				
GO:0005783~endoplasmic reticulum	6	13.72%	0.003	FT, SPX2, YUC4, PDIL1-4, PHO2, MSRB3
**Molecular Function**				
GO:0016810~hydrolase activity, acting on carbon–nitrogen (but not peptide) bonds	2	4.57%	0.020	NIT1, AMI1
**KEGG analysis**	**Count**	**Percent of Proteins**	***p*-Value**	**Genes**
**ath00500:Starch and sucrose metabolism**	4	9.15%	0.007	DPE2, SS2, TPS3, SUS1

**Table 3:** Trehalose-6-phosphate synthase gene in rice samples.

Genes	Gene Symbol	Gene Name	logFC	FDR
LogFC < 0				
Os11t0513900-01	OsTPS8, OsTPS1, TPS1	trehalose-6-phosphate synthase 1	−1.589154	0.5285901
Os02t0790500-02	OsTPS5	trehalose-6-phosphate synthase 5	−0.425324	0.08463762
Os11t0156600-00	OsTPS8, OsTPS1, TPS1	trehalose-6-phosphate synthase 1	−0.3653828	0.9241036
Os08t0409100-01	OsTPP6	trehalose-6-phosphate phosphatase 6	−0.141309	0.6490201
Os03t0372500-03	OsTPP1, OsTPS	trehalose-6-phosphate phosphatase 1, trehalose 6-P synthase	−0.000663483	0.9997236
LogFC > 0				
Os09t0332100-01	OsTPP3	trehalose-6-phosphate phosphatase 3	0.2046861	0.7819962
Os09t0369400-01	OsTPP7	trehalose-6-phosphate phosphatase 7	0.3268572	0.4806699
Os10t0553300-01	OsTPP2	trehalose-6-phosphate phosphatase 2	0.368067102	0.601752321
Os03t0386500-01	OsTPP9	trehalose-6-phosphate phosphatase 9	0.7412287	0.04455191
Os02t0753000-01	OsTPP4	trehalose-6-phosphate phosphatase 4	1.243797	0.318923

## Data Availability

Data is contained within the article or Supplementary Materials.

## Supplementary Materials

The following are available online at https://www.mdpi.com/article/10.3390/genes12050641/s1, Supplementary Table S1: The location of T-DNA insertions over the rice chromosomes; Supplementary Table S2: Raw data information; Supplementary Table S3: Alignment rate; Supplementary Table S4: Primers used in qRT-PCR for *PsGAPDH* and the validation of *trehalose-6-phosphate synthase* genes.
