# Supplementary material for: Transcriptomic Analysis of Rice Plants Overexpressing PsGAPDH in Response to Salinity Stress
Source: Genes (Basel). 2021 Apr 25;12(5):641. doi: 10.3390/genes12050641 (PMC8146104; doi:10.3390/genes12050641)
Supplement: Supplementary file 1 [file genes-12-00641-s001.zip › genes-1180213-SI.pdf]

Table S1: The Location of T-DNA insertions over the rice chromosomes

No	Line	Insertion Type	Chromosome	Gene_ID	Description
1	#2-2	5'Upstream-1000	chr03	Os03t0244700-02 upstream 0.866kb	Armadillo-like helical domain containing protein.
**2**	**#5-2**	**Intergenic**	chr02	Os02t0682200-01 upstream 10.024kb	Similar to MADS box protein.
**3**	**#6-1**	**Intergenic**	chr07	Os07t0675166-01 upstream 9.238kb	Non-protein coding transcript.
**4**	**#10-1**	**Intergenic**	chr08	Os08t0203150-00 upstream 1.413kb	Hypothetical gene.
5	#12-2	5'Upstream-1000	chr06	Os06t0681950-00 upstream 0.829kb	Hypothetical conserved gene.
6	#13-1	3'Downstream-300	chr05	Os05t0494200-02 downstream 0.22kb	Similar to Cysteine proteinase inhibitor-II (Oryzacystatin-II).
7	#14-1	Intergenic	chr09	Os09t0348766-01 upstream 15.858kb	Hypothetical gene.
**8**	**#17-2**	**Intergenic**	chr10	Os10t0130701-01 upstream 1.231kb	Hypothetical conserved gene.
9	#19-2	3'Downstream-300	chr02	Os02t0730300-01 downstream 0.04kb	Similar to Potassium transporter HAK3p (Fragment).
10	#21-2	5'Upstream-1000	chr06	Os06t0681950-00 upstream 0.966kb	Hypothetical conserved gene.

Table S2: Raw data information

Sample Name	Read Length	Total Base	Read Count	GC	Q20Ratio	Q30Ratio
1-NW	100	1,088,687,500	10,886,875	53	0.988	0.939
2-NW	100	930,753,100	9,307,531	53	0.987	0.934
3-NG1	100	917,845,000	9,178,450	53	0.987	0.932
5-NG1	100	1,091,399,300	10,913,993	53	0.987	0.934
4-NG2	100	1,125,825,700	11,258,257	53	0.989	0.939
8-NG2	100	1,006,104,400	10,061,044	54	0.986	0.931
6-NG3	100	939,689,900	9,396,899	53	0.987	0.933
9-NG3	100	1,021,055,600	10,210,556	53	0.987	0.930
7-SW	100	924,198,300	9,241,983	53	0.987	0.932
23-SW	100	1,049,474,300	10,494,743	53	0.988	0.941
12-SG1	100	1,099,991,100	10,999,911	53	0.989	0.943
27-SG1	100	938,350,000	9,383,500	53	0.987	0.935
10-SG2	100	906,256,000	9,062,560	52	0.987	0.935
13-SG2	100	1,138,778,900	11,387,789	54	0.988	0.943
11-SG3	100	1,121,420,300	11,214,203	53	0.987	0.938
14-SG3	100	1,258,748,600	12,587,486	53	0.988	0.944

Table S3: Alignment rate

**SampleName**	**OverallAlignmentRate**	**ConcordantZero**	**ConcordantPairAlignment**	**MultipleAlignment**
1-NW	98.49%	3.10%	94.26%	2.65%
2-NW	98.73%	2.63%	81.93%	15.44%
3-NG1	97.76%	4.10%	93.21%	2.70%
5-NG1	97.90%	3.95%	82.05%	14.00%
4-NG2	98.30%	3.06%	65.60%	31.34%
8-NG2	97.99%	3.57%	93.57%	2.86%
6-NG3	98.15%	3.37%	71.27%	25.37%
9-NG3	97.81%	4.12%	93.13%	2.75%
7-SW	98.69%	2.76%	83.07%	14.17%
23-SW	98.59%	2.97%	93.87%	3.16%
12-SG1	97.81%	3.92%	77.55%	18.53%
27-SG1	97.95%	3.82%	92.61%	3.57%
10-SG2	97.94%	3.87%	93.22%	2.91%
13-SG2	97.53%	4.30%	89.54%	6.16%
11-SG3	97.53%	4.52%	91.84%	3.64%
14-SG3	98.17%	3.27%	71.67%	25.07%

Table S4: Primers used in qRT-PCR for PsGAPDH and validation of trehalose-6-phosphate synthase genes

No	Target gene	Primer sequences (5′→3′)
1	*PsGAPDH*	F: AAGCCCGCTGATTACAAAGAGA<br>R: GGCGAGAGTATCCGTATTCGTT
2	Os11t0513900-01 (*OsTPS8, OsTPS1, TPS1*)	F: CACATGAGCGACGAGCTAAAC<br>R: TCATCCACACGGAAGAAACCT
3	Os02t0790500-02 (*OsTPS5*)	F: GAAAGCGCGTTAGTTTGGCA<br>R: TGGCCCCTCTTAACAACGAC
4	Os08t0409100-01 (*OsTPS6*)	F: ACCCCAAACTGAAGCTCACG<br>R: GAACCCTAGCGACTCGAGGA
5	Os09t0332100-01(*OsTPP3*)	F: GGTTGTTTGGCGGGACCTAC<br>R: GGGGTTGTTGTGCTGGTAGTA
6	Os03t0386500-01 (*OsTPP9*)	F: CATGACGGACGAGATGAGGG<br>R: CTTGGATGTCCATCCCGTGG
7	Os02t0753000-01 (*OsTPP4, OsTPP7*)	F: CAAGGTTCGCAACTTCGTCG<br>R: CGATCATGGGGAGGAACTCG
8	*OsActin1*	F: ACAGGTATTGTGTTGGACTCTGG<br>R: AGTAACCACGCTCCGTCAGG
